# HIV-1 Tat induces DNMT over-expression through microRNA dysregulation in HIV-related non Hodgkin lymphomas

**DOI:** 10.1186/1750-9378-9-41

**Published:** 2014-12-09

**Authors:** Anna Luzzi, Federica Morettini, Sara Gazaneo, Lucia Mundo, Anna Onnis, Susanna Mannucci, Emily A Rogena, Cristiana Bellan, Lorenzo Leoncini, Giulia De Falco

**Affiliations:** Department of Medical Biotechnologies, University of Siena, Siena, Italy; Department of Pathology, University of Nairobi, Nairobi, Kenya; School of Biological and Chemical Sciences, Queen Mary University of London, London, UK

**Keywords:** HIV, Aggressive B-cell lymphomas, microRNAs, DNMTs, Tat

## Abstract

**Background:**

A close association between HIV infection and the development of cancer exists. Although the advent of highly active antiretroviral therapy has changed the epidemiology of AIDS-associated malignancies, a better understanding on how HIV can induce malignant transformation will help the development of novel therapeutic agents.

**Methods:**

HIV has been reported to induce the expression of DNMT1 *in vitro*, but still no information is available about the mechanisms regulating DNMT expression in HIV-related B-cell lymphomas.

In this paper, we investigated the expression of DNMT family members (DNMT1, DNMT3a/b) in primary cases of aggressive B-cell lymphomas of HIV-positive subjects.

**Results:**

Our results confirmed the activation of DNMT1 by HIV *in vivo*, and reported for the first time a marked up-regulation of DNMT3a and DNMT3b in HIV-positive aggressive B-cell lymphomas. DNMT up-regulation in HIV-positive tumors correlated with down-regulation of specific microRNAs, as the miR29 family, the miR148-152 cluster, known to regulate their expression. Literature reports the activation of DNMTs by the human polyomavirus BKV large T-antigen and adenovirus E1a, through the pRb/E2F pathway. We have previously demonstrated that the HIV Tat protein is able to bind to the pocket proteins and to inactivate their oncosuppressive properties, resulting in uncontrolled cell proliferation. Therefore, we focused on the role of Tat, due to its capability to be released from infected cells and to dysregulate uninfected ones, using an *in vitro* model in which Tat was ectopically expressed in B-cells.

**Conclusions:**

Our findings demonstrated that the ectopic expression of Tat was *per se* sufficient to determine DNMT up-regulation, based on microRNA down-regulation, and that this results in aberrant hypermethylation of target genes and microRNAs.

These results point at a direct role for Tat in participating in uninfected B-cell lymphomagenesis, through dysregulation of the epigenetical control of gene expression.

## Background

A close association between Human Immunodeficiency Virus (HIV) infection and the development of a number of cancers, NHL being the second most common, has been described. Interestingly, AIDS-associated lymphomas are of B-lymphoid origin in at least 95% of all cases described despite the fact that HIV infects T-lymphocytes [1], raising the question whether HIV may have a direct role in B-cell lymphomagenesis. To date, there are no clear answers to explain how HIV leads to transformation, even though several events have been proposed as co-factors in HIV-related tumorigenesis. The frequencies of different subpopulations of B-cells have been reported altered in the presence of HIV Reviewed in [2]. These changes include increased frequency of activated and terminally differentiated B-cells expressing low levels of CD21 that have been associated with ongoing viral replication [3, 4], a decreased frequency of memory B-cells that is not reversed by antiretroviral therapy [5], and an increased frequency of immature/transitional B-cells that has been associated with CD4^+^ T-cell lymphopenia [6–8]. Although there is no indication of a direct role for the virus in the B-cell transformation, lymphadenopathy, polyclonal B-cell proliferation, and even lymphoma may precede overt compromise of T-cell immunity [9, 10]. In addition, changes in the microenvironment of the host cells have been recorded following HIV infection [11], as well as chronic immune activation and dysfunctional cytokine production that have been described throughout all stages of HIV-1 infection [12].

In addition to these indirect effects, HIV may directly contribute to B-cell transformation through its encoded proteins and/or viral microRNAs (miRNAs), using which it can disturb gene and miRNA expression in host cells [13]. It is noteworthy that most transformed B-cells do not contain the virus, therefore some other mechanisms and/or viral factors may contribute to transformation. In particular, several findings support an oncogenic role of the HIV-1 Tat protein, which is essential for viral gene expression and virus production [14–16]. A soluble, biologically active form of Tat is released by HIV-infected cells, taken up and translocated to the nucleus by neighbouring uninfected ones [17–19], and may directly contribute to B-cell abnormalities in HIV-positive patients [20]. We have previously demonstrated that B-cell lymphomas of HIV-infected individuals may be positively stained by an anti-Tat antibody [21]. Therefore it is reasonable to hypothesize that the endocytosed Tat, released from infected cells, may then exert its pleiotropic activities in uninfected B-cells. Tat has been reported to modulate the expression of several cellular genes, including cytokines and their receptors [22–24]. In particular, the ability of Tat to increase the expression of interleukins-6 (IL-6) and 10 (IL-10) [25, 26], which in turn promote B-cell stimulation, and the evidence that about 30% of Tat-transgenic mice develop B-cell lymphomas [27], suggest that Tat might play a role in the pathogenesis of HIV-related B-lymphomas. In particular, IL-6 has been reported to induce the over-expression of the DNA Methyltransferase 1 (DNMT1), which has a key role in the maintenance of DNA methylation, and epigenetically regulate the expression of several genes, in liver cancer through miRNA dysregulation, which correlates with increased genomic methylation [28]. Interestingly, HIV has been reported to induce the expression of DNMT1 [29, 30]*in vitro*, though there is no evidence that this can be exerted through IL-6 in HIV-positive individuals, although serum IL-6 is significantly elevated in HIV^+^ subjects who develop aggressive B-cell lymphomas [31]. In addition, induction of DNMT aberrant activity has been reported by several human viruses through the pRb/E2F pathway [32]. In particular, this occurs through the interaction of viral products with the RB proteins and their consequent inactivation [33–37]. Noteworthy, we have previously reported the physical interaction of Tat with the pocket proteins, which results in their inactivation and inhibition of their growth regulatory properties [21, 38]. This suggests that Tat may contribute to DNMT aberrant expression in HIV-positive subjects.

In this paper we have investigated the possible mechanisms used by HIV to induce DNMT over-expression. In particular, we have analyzed whether DNMT induction by HIV could depend on specific miRNA dysregulation, as reported in liver cancer [28, 39]. Our results show that DNMT1, DNMT3a/b are up-regulated in B-cell lymphomas, and that this relies on down-regulation of specific miRNAs. To assess the possible contribution of Tat, we used an *in vitro* model, in which Tat was ectopically expressed in uninfected B-cells. The ectopic expression of Tat resulted in the up-regulation of DNMT1, DNMT3a/b based on down-regulation of specific miRNAs, in accordance to what we observed in HIV-positive primary tumors.

DNMT over-expression may result in altered methylation pattern of genes and/or microRNAs, therefore we investigated whether it may affect the expression of genes frequently reported to be inactivated by hypermethylation, as *INK4/p16*, *TP53* and *RB1*. In addition, we tested whether down-regulation of DNMT-regulating miRNAs detected in our cell model was possibly dependent on hypermethylation as well, in a feedback-loop mechanism fashion. Here we show that the ectopic expression of Tat determines an altered methylation pattern of *INK4/p16* and of specific miRNAs, this finding being also confirmed in HIV-positive tumors.

These results point out at the possible role for Tat in participating in B-cell lymphomagenesis in uninfected cells, through dysregulation of the host cell miRNA machinery and of the epigenetic control of gene expression, and provide novel information to the molecular mechanisms of B-cell lymphomagenesis in HIV-infected individuals.

## Methods

### Ethics statement

The Institutional Review Board of the University of Siena (Italy) and the Ethics and Research Committee of the University of Nairobi (Kenya) gave ethics approval for this study. Informed written consent was obtained in all cases.

### Case selection and immunophenotype

For this study aggressive 30 formalin-fixed paraffin-embedded (FFPE) cases of HIV-positive B-cell lymphoma (DLBCL, BL) and 30 formalin-fixed paraffin-embedded cases of HIV-negative B-cell lymphoma (DLBCL, BL) collected at the Department of Pathology, Nairobi Hospital, Kenya and the Department of Human Pathology and Oncology, University of Siena, Italy, have been used. Cases were reviewed by expert pathologists (BC, LL) and diagnoses were confirmed by morphology on histological slides stained with HE, Giemsa and by immunophenotyping, according to the Word Health Organization (WHO) [1]. 5 reactive lymph nodes were used as negative controls. Immunohistochemical studies were performed on representative paraffin sections from each case using microwave pre-treatment of slides for antigen retrieval, as previously reported [40]. A large panel of antibodies recognizing formalin-resistant epitopes of the various antigens was applied (Table 1). The presence of the Epstein-Barr virus (EBV) was assessed by *in situ* hybridization for EBERs as described [41]. HIV-positive cases were mostly positive for EBV.

**Table 1:** **List of the antibodies used for immunohistochemistry**

Primary antibody	Dilution	Company
DNMT-1	1:50	BD
DNMT3A	1:50	Abcam
TAT	1:100	Abcam
BCL6	1:30	Dako
BCL2	1:150	Dako
CD20	1:150	NeoMarkers
IgM	1:10000	Dako
CD30	1:50	NeoMarkers
CD10	1:20	NeoMarkers
CD79	1:50	NeoMarkers
Irf-4	1:50	Dako
CD38	1:100	Dako

### PCR for detection of HIV infection

All of the HIV-positive lymphomas were tested for HIV genome presence. A fragment of the HIV-1 DNA was amplified by nested PCR using the lentivirus universal primer pair UNIPOL1/2 as outer primers (25 cycles) and the degenerate primers UNIPOL3 (50-GAAACAGGAMRRGAGACAGC-30) and UNIPOL4 (50-TTCATDGMTTCCACTACTCCTTG-30) as inner primers (30 cycles) [42]. This nested primer set, when used at low-stringency annealing, specifically amplifies all HIV-1 and HIV-2 pol sequences known to date. PCR products were visualized on agarose gels and the specificity of the products was confirmed by direct sequencing.

### Computational analysis

miRNAs predicted to regulate the expression of DNMT1 (hsa-miR-130a, hsa-miR-130b, hsa-miR-148a, hsa-miR-148b, hsa-miR-152, hsa-miR-301) and DNMT3a/b (hsa-miR-29a, hsa-miR-29b and hsa-miR-29c, hsa-miR-148a, hsa-miR-148b) were identified by computational analysis, using web-available resources (Mirnaviewer, PicTar, Tarbase [43] and miRBase [44]; mirnaviewer is available at http://cbio.mskcc.org/mirnaviewer; PicTar is a project of the Rajewsky lab at NYU's Center for Comparative Functional Genomics and the Max Delbruck Centrum, Berlin). Among the many available by bioinformatics predictions, these specific miRNAs were selected for this study as regulation of DNMTs by these miRNAs through direct mRNA binding has been previously proved [45, 46].

### MiRNA extraction

Extraction of miRNAs from FFPE sections of primary tumors and reactive lymph nodes was performed using the miRNA easy FFPE kit (Qiagen, Carlsbad, CA), following manufacturer’s instructions. Quality and purity of RNA were assessed by spectrophotometric read using Nanodrop (Thermo Scientific, Wilmington, DE) and by Agilent Bioanalyzer (Agilent Technologies, Santa Clara, CA).

### Analysis of miRNA expression

MiRNA expression was analyzed by RT-qPCR as previously described [41]. For each sample, 10 ng of total RNA were reverse transcribed. Real-time PCR was performed using Taqman probes specific for each miRNA (hsa-miR-130a, hsa-miR-130b, hsa-miR-148a, hsa-miR-148b, hsa-miR-152, hsa-miR-301, hsa-miR-29a, hsa-miR-29b and hsa-miR-29c), and for RNU43, used as an endogenous control (Applied Biosystems, Applera, Italy). Amount and quality of RNA were evaluated measuring the OD at 260 nm, the 260/230 and the 260/280 ratios by Nanodrop (Celbio, Italy).

### Gene expression analysis

Relative quantification of gene expression for *Cyclin A*, *DNMT1*, *DNMT3a*, *DNMT3b, INK4/p16, RB1 and TP53* was also carried out by Real-time PCR using FluoCycle SYBR green (Euroclone, Celbio, Italy) according to manufacturer’s instructions. *HPRT* was used as housekeeping gene. The complete list of primers used for qPCR is provided in Table 2. Differences in gene expression were calculated using the ΔΔCt method [47].

**Table 2:** **Primers used for qPCR**

Gene	Primer sequence
*CYCLIN A-*FORWARD	5’-AGG CTT CAA AGT ACC TGT GTG-3’
*CYCLIN A-*REVERSE	5’-TTG ATC CCA CGT GCA GAA G-3’
*DNMT1*-FORWARD	5’-CGACTACATCAAAGGCAGCAACCTG-3’
*DNMT1*-REVERSE	5’-TGGAGTGGACTTGTGGGTGTTCTC-3’
*DNMT3A*-FORWARD	5’-TAT TGA TGA GCG CAC AAG AGA GC-3'
*DNMT3A*-REVERSE	5’-GGG TGT TCC AGG GTA ACA TTG AG-3'
*DNMT3b*-FORWARD	5’-GGC AAG TTC TCC GAG GTC TCTG-3'
*DNMT3b*-REVERSE	5’-TGG TAC ATG GCT TTT CGA TAG GA-3'
*RB1* FORWARD	5’-CAC CAA TAC CTC ACA TTC CTC-3'
*RB1* REVERSE	5’-TTC TCA GAA GTC CCG AAT G-3’
*TP53* FORWARD	5’-CCA TCC TCA CCA TCA TCA C-3'
*TP53* REVERSE	5’-GGC AGT GCT CGC TTA GTG G-3'
*INK4/p16* FORWARD	5'-GGA AGG TCC CTC AGA CAT C-3'
*INK4/p16* REVERSE	5’-GCA GTT GTG GCC CTG TAG-3'

Primers for *CYCLIN A* amplified a region of 105 bp; Primers for *DNMT1* amplified a region of 88 bp; Primers for *DNMT3a* amplified a region of 68 bp; Primers for *DNMT3b* amplified a region of 68 bp; Primers for *RB1* amplified a region of 152 bp; Primers for *TP53* amplified a region of 140 bp; Primers for *INK4/p16* amplified a region of 67 bp.

### Recombinant Tat

The recombinant Tat HIV-1 IIIB (aa 1–86) from Dr J Raina was obtained through the EU Programme EVA/MRC Centralised Facility for AIDS Reagents, NIBSC, UK (Grant numbers QLK2-CT-1999-00609 and GP828102). The stock solution was diluted in saline citrate buffer as recommended, and aliquots were stored at -80°C until use. The concentration of endotoxin was below 0.01 endotoxin unit (EU)/mg of protein. Extracellular Tat (50 ng/ml) was added to the medium culture of cells for 48 h. Cells grown in the absence of Tat were used as a control.

### Ectopic expression of Tat *in vitro*

Tat ectopic expression was either obtained through exposure to recombinant Tat or through transient and stable transfections, by nucleofection. A Burkitt lymphoma-derived EBV-negative cell line (Ramos) was used to perform the *in vitro* experiments. Briefly, cells were cultured in RPMI supplemented with 10% FBS, 1% L-glutamine, penicillin/streptomycin, with 5% CO_2_, at 37°C. The recombinant Tat was used as previously described [38]. Cells grown in the absence of Tat were used as a negative control. Transient and stable transfections were performed by nucleofection, using an Amaxa apparatus, program T16 and solution T (Amaxa, Cologne, Germany). A transfection efficiency of 45% was obtained, as assessed by FACS analysis for a GFP reporter. Cells (2x10^6^) were transfected with 10 μg of pCDNA3-Tat [38], using the empty vector as negative control. In addition, a stable Tat-transfected Ramos cell line was obtained by antibiotic selection with G-418, at the concentration of 2 mg/ml.

### Cell proliferation

The effect of Tat on cell proliferation was assessed as previously described [40]. Briefly, cell counts were established by Tripan blue staining and the possible effect of Tat on the cell cycle was monitored by analysis of Cyclin A expression, used as a specific S-phase marker, by RT-qPCR, as described above. In addition, proliferation was also monitored following transfections with miRNA mimics and inhibitors (see below). Statistical significance was assessed by the analysis of variance (ANOVA) test.

### miRNA nucleofection

To assess the regulation of the target genes by the predicted miRNAs in our cell model, modulation of the endogenous miRNAs was obtained by synthetic miRNAs and inhibitors (Dharmacon, Celbio, Milan, Italy), through nucleofection, followed by detection of the expression of the genes of interest. Briefly, cells were split the day before nucleofection and 5x10^6^ cells were transfected with different concentrations of either the miRNA mimic or inhibitor (10 nM, 50 nM or 100 nM), to assess the best dose–response concentration. Negative control of mimics and inhibitors (NC, NCI, respectively) were used at the 10 nM concentration (Dharmacon, Euroclone, Milan, Italy). As the selected miRNAs regulating DNMT1 map in clusters, we used one mimic/inhibitor for each cluster. In particular, mimics and inhibitors of hsa-miR130a, hsa-miR152 and hsa-miR29 were used to modulate the endogenous expression of DNMT1 and DNMT3a/b, respectively (all from Dharmacon, Euroclone, Milan, Italy). To analyze the downstream DNMT modulation, mimics and inhibitors were used at the concentration which gave the best effect. RNA was extracted 24 hours after nucleofection and both gene expression for DNMTs and miRNA expression were checked by Real-Time RT-PCR, as previously described.

### Western blotting

Cells pellets were lysed on ice for in EBC buffer (50 nM Tris–HCl pH 8.0, 130 mM NaCl, 1% Triton X-100, 0.1% SDS) supplemented with protease inhibitor cocktail (Sigma, Milan-Italy). Cell lysates were separated by 10% SDS-PAGE gel followed by transfer to Hybond ECL nitrocellulose membrane (GE Healthcare, Milan, Italy). Western blotting was made using anti-DNMT1 (1:400, BD, NJ USA), anti-DNMT3a (1:250, Abcam, UK) and anti-actin (1:1000, BD, NJ USA). Secondary antibodies conjugated with HRP were used at a dilution of 1:5000 and the reaction was revealed using the ECL Western Blotting Kit (Promega, Milan-Italy) according to the manufacturer’s instructions.

### DNA extraction and methylation assay

FFPE section (10 μm) of 5 cases of DLBCL HIV-positive, 5 cases of DLBCL HIV-negative and 2 reactive lymph nodes were deparaffinized with xylene and DNA extraction was performed with NucleoSpin kit (Macherey-Nagel), according to manufacturer’s instructions. Amount and quality of DNA were evaluated measuring the OD at 260 nm, the 260/230 and the 260/280 ratios by Nanodrop (Celbio, Italy). DNA quality control PCR was also performed as previously described (21). 500 ng of DNA of each case were modified by bisulfite (EZ DNA Methylation-Gold kit ZYMO RESEARCH) according to manufacturer’s instructions. Approximately 100 ng of converted DNA were amplified using methylation specific primers (MSP) or bisulfite sequencing primers (BSP). Primer sequences for MSP are provided in Table 3. PCR products were separated on a 2% agarose gel to confirm size. For methylation assay primers were designed using the MethPrimer (MethPrimer *- Li Lab, UCSF*http://www.urogene.org/methprimer/index1.html). For some microRNAs the software did not identify any CpG islands (hsa-miR130b, hsa-miR148b, hsa-miR301, hsa-miR-29a, hsa-miR-29b, hsa-miR-29c), therefore methylation was assessed for miRNAs containing CpG islands. In some cases, designed primers amplified a product size which was too long to be amplified in our primary tumors, according to the DNA quality control, and therefore they were discarded. Methylation was therefore checked using different approaches, collectively MSP and BSP, followed by direct sequencing, and treatment with 5-Aza. Methylation assay was carried out either in primary tumors or Tat-transfected cell lines by MSP and BSP, according to the product size, and by 5-Aza treatment in cell lines (see below). Primer sequences are reported in Table 4.

**Table 3:** **Primer sequences for MSP**

Gene	Primer sequence
*INK4/p16* U FORWARD	5’-TTA TTA GAG GGT GGG GTG GAT TGT-3’
*INK4/p16* U REVERSE	5’-CAA CCC CAA ACC ACA ACC ATA A-3’
*INK4/p16* M FORWARD	5’-TTA TTA GAG GGT GGG GCG GAT CGC-3’
*INK4/p16* M REVERSE	5’-GAC CCC GAA CCG CGA CCG TAA-3’
*TP53* U FORWARD	5’-TTT TTT AGG TAG TTT TTG GTT TTG T-3’
*TP53* U REVERSE	5’-ACC AAA CCT CTC AAA TTA CAA CAA T-3’
*TP53* M FORWARD	5’-ATT TTT TTA GGT AGT TTT CGG TTT C-3’
*TP53* M REVERSE	5’-GAA CCT CTC AAA TTA CGA CGA T-3’

Primers for *INK4/p16* unmethylated (U) amplified a region of 151 bp, primers for *INK4/p16* methyled (M) amplified a region of 150 bp; primers for *TP53* unmethylated amplified a region of 134 bp, primers for *TP53* methylated amplified a region of 133 bp.

**Table 4:** **Primer sequences for microRNA methylation analysis**

microRNA	Primer sequence
hsa-miR148a FORWARD (BSP)	5’-TGGGTATTTGTTTTTGTTGATTG-3’
hsa-miR148a REVERSE (BSP)	5’ACTACACTTAAACCCCCTCTAACC-3’
hsa-miR152 FORWARD (BSP)	5’-GGAATTTTGTGTTATTTTTGATTG-3’
hsa-miR152 REVERSE (BSP)	5’CCAAACAAATATTCCACTACAAAC-3’
hsa-miR148a U-FORWARD (MSP)	5’TTGATGTTGTTAGTGTTTTAGATGT-3’
hsa-miR148a U-REVERSE (MSP)	5’AAAAAAATTAATCAAATTCCTCATA-3’
hsa-miR148a M-FORWARD (MSP)	5’GTCGATGTCGTTAGTGTTTTAGAC-3’
hsa-miR148a M-REVERSE (MSP)	5’AAAAAAATTAATCAAATTCCTCGTA-3’

hsa-miR148a hsa-miR148a (BSP) amplify a region of 326 bp; hsa-miR152 (BSP) amplify a region of 435 bp. Appropriate primers for MSP were found by the software only for hsa-miR148a, which amplify a region of 110 bp.

### Treatment with 5-aza-2-deoxycitidine

To test whether miRNA down-regulation in HIV-positive tumors was possibly due to hypermethylation following DNMT over-expression, Ramos cells (either transfected or not with a vector coding for Tat) were treated with 1 μM 5-aza-2-deoxycitidine as reported [41] and relative quantification of miRNAs was made by RT-qPCR two days after treatment, as previously described.

## Results

### HIV induces the aberrant expression of DNMTs

**Figure 1:**
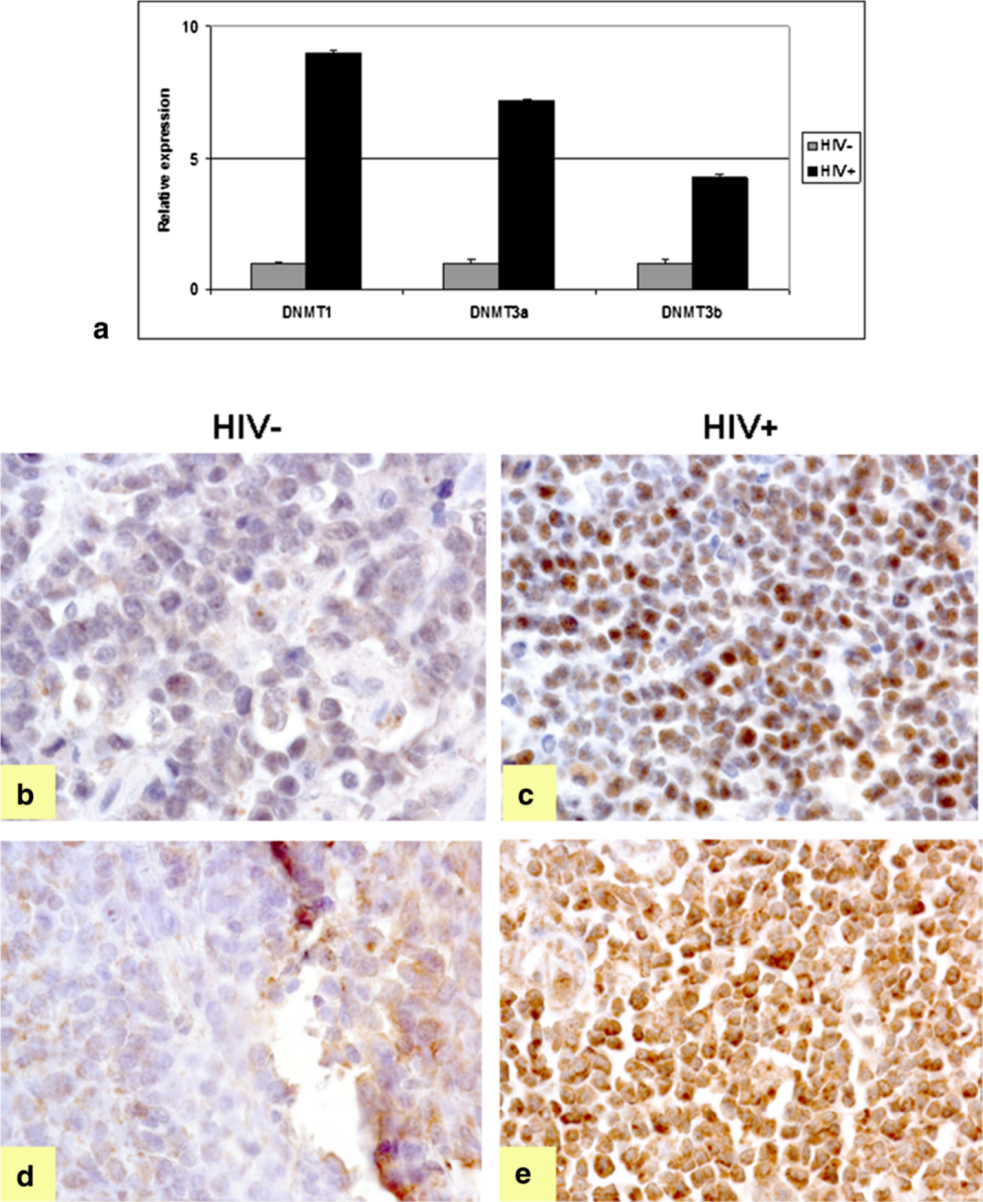
**Expression of DNMTs in HIV** ^**+**^ **vs. HIV** ^**-**^ **tumors. (a)** Relative expression of DNMT1, DNMT3a/b in HIV^+^ vs. HIV^-^ tumors by RT-qPCR. Up-regulation of all these genes is observed in HIV^+^ samples (p < 0.05). **(b-c)** IHC using an anti-DNMT1 antibody in HIV-negative cases (b) and HIV-positive cases (c). **(d-e)** IHC for DNMT3a in HIV-negative **(d)** and positive **(e)** cases. Up-regulation of DNMT1 and DNMT3a was detected in HIV-positive tumors. None of the antibodies tested for DNMT3b was useful for IHC.

HIV is reported to induce the expression of DNMT1 *in vitro*[29, 30], though no information is available about its effect on DNMT3a/b, and about their expression in HIV-positive B-cell lymphomas. Therefore relative expression of DNMTs was checked in HIV-positive and HIV-negative primary tumors of aggressive B-cell lymphomas vs. reactive lymph nodes, both by RT-qPCR (Figure 1a) and immunohistochemistry (Figure 1b-e), respectively. DNMT1, DNMT3a/b were found up-regulated in HIV-positive tumors (Figure 1a-e).

**Figure 2:**
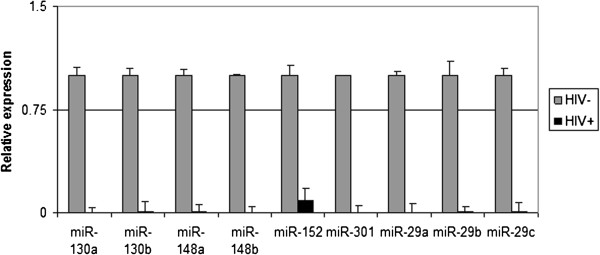
**miRNAs predicted to regulate DNMT1, DNMT3a/b were analyzed by RT-qPCR in HIV** ^**+**^ **vs. HIV** ^**-**^ **primary tumors.** A strong and significant down-regulation of all the selected miRNAs regulating DNMTs is observed in HIV-positive tumors (p < 0.05). The graph is representative of three different RT-qPCR experiments. Error bars represent standard deviation between duplicates.

As DNMT dysregulation in cancer has been linked to dysregulation of miRNAs [28, 39, 45, 48, 49], the expression of miRNAs known to directly regulate DNMTs was investigated. Six miRNAs (hsa-miR-130a, hsa-miR-130b, hsa-miR-148a, hsa-miR-148b, hsa-miR-152, hsa-miR-301) have been reported to regulate the expression of DNMT1 [28], whereas the miR29 family and also hsa-miR148 regulate DNMT3 [45, 46]. Our results demonstrate a marked down-regulation of all the selected DNMT-regulating miRNAs in HIV-positive tumors (Figure 2), in respect with HIV-negative samples and normal lymph nodes.

### DNMT expression is increased in Tat-transfected cells

**Figure 3:**
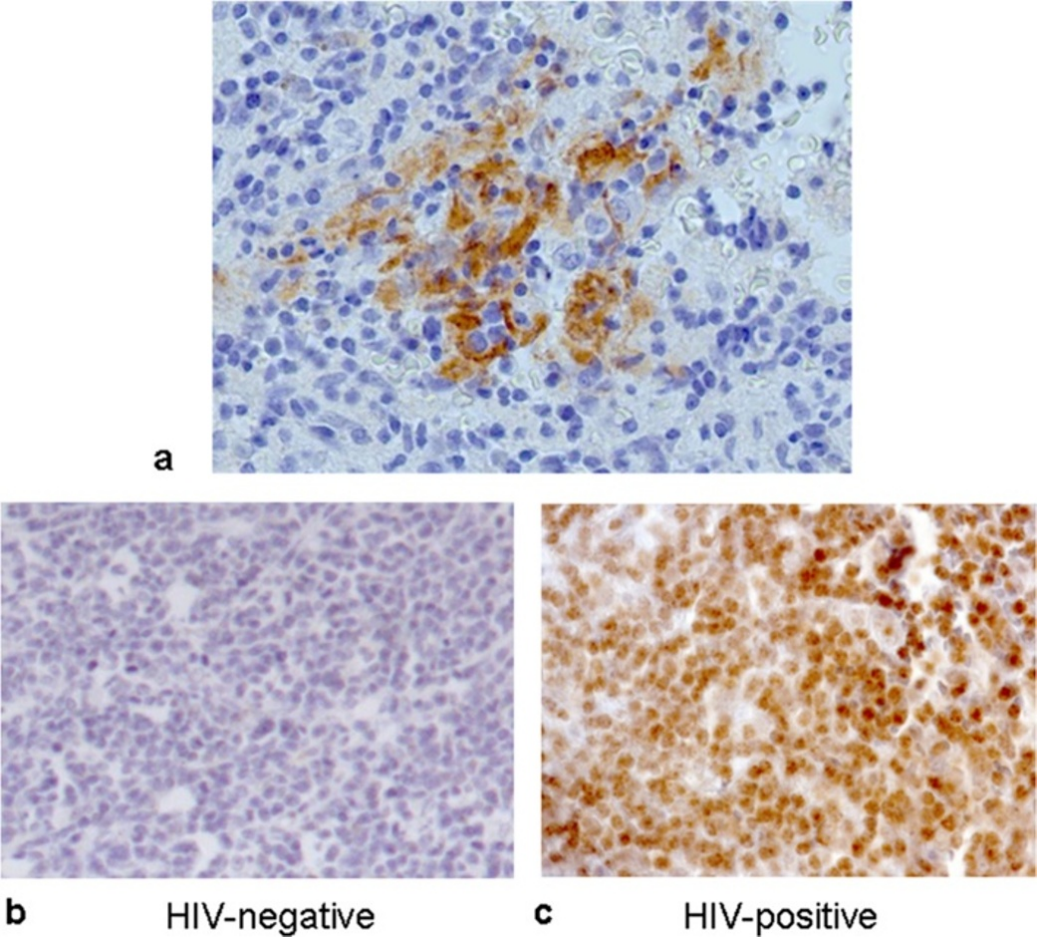
**Immunoistochemical staining for p24 and Tat in HIV** ^**+**^ **vs. HIV** ^**-**^ **tumors. (a)** IHC using an anti-p24 antibody showed marked positivity of macrophages in a B-cell lymphoma of a HIV-positive subject. **(b-c)** IHC using an anti-Tat antibody in HIV-negative **(b)** and HIV-positive cases **(c)**. Marked positivity to Tat of uninfected B-cells is visible in HIV-positive tumors, thus indicating that a soluble form of the protein is released from infected cells and enters neighbouring uninfected ones.

**Figure 4:**
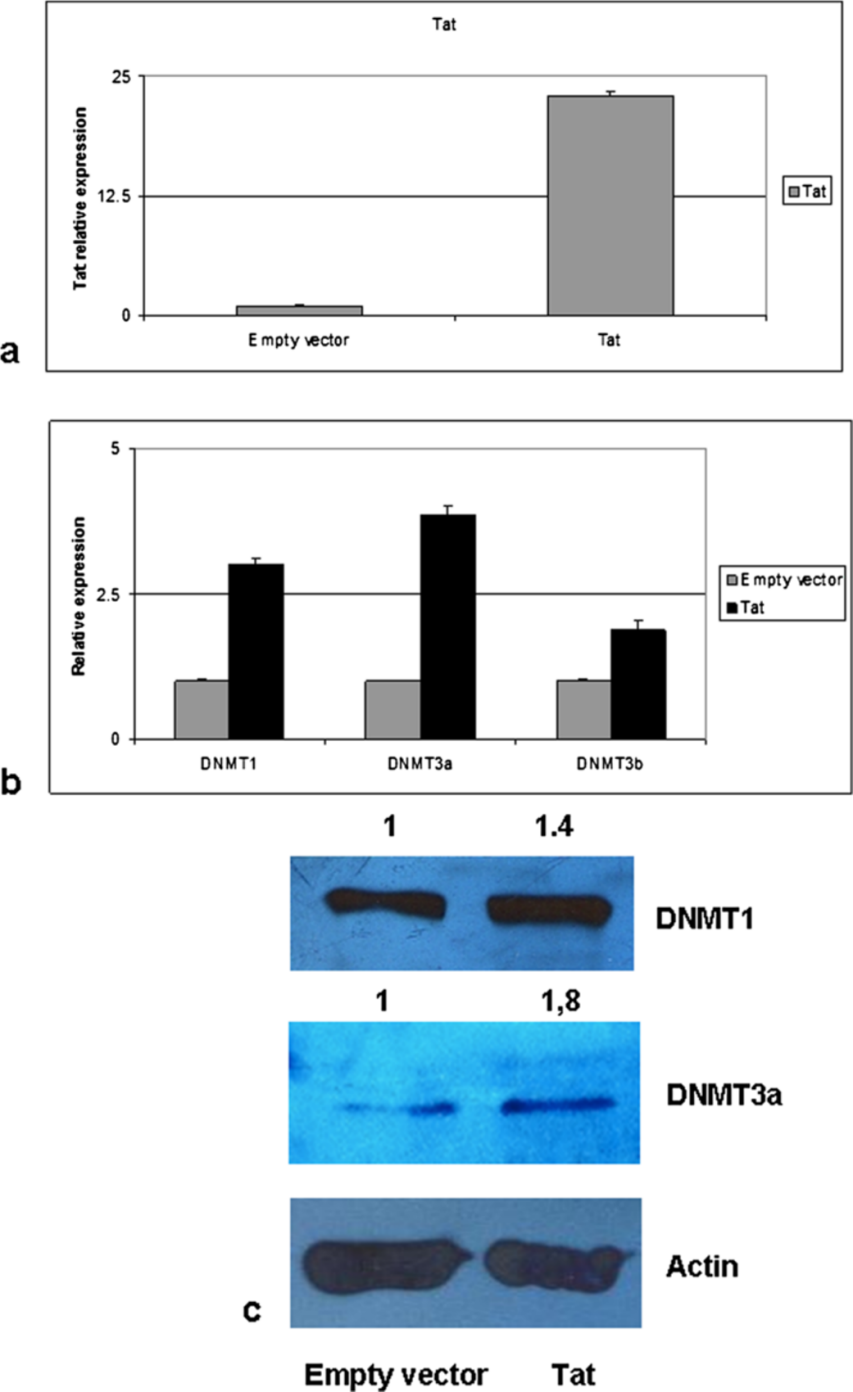
**DNMT expression in Tat-positive vs. Tat-negative cells. (a)** Relative expression of Tat 24 h after transfection with a Tat-coding vector. **(b-c)** Ectopic expression of Tat was obtained either upon exposure to the recombinant protein or following transfection of a Tat-coding vector. DNMT1, DNMT3a/b expression was then analysed in Tat-positive (transfected or treated with soluble Tat) vs. Tat-negative cells (empty vector-transfected or untreated cells) at the mRNA level by RT-qPCR **(b)**; up-regulation of all DNMTs is obtained following Tat ectopic expression, both upon exposure to recombinant Tat and following Tat-transfection (p < 0.05). **(c)** Western blotting analysis for DNMT1 and DNMT3a in Tat-positive vs. Tat-negative cells. Up-regulation of both DNMTs is observed in Tat-positive cells, thus confirming results obtained in HIV-positive primary tumors. None of the tested antibodies for DNMT3b was suitable for WB analysis. Quantification by densitometric analysis is reported.

We tested tumor tissues for the presence of HIV, which was confirmed in our series of cases by p24 staining by IHC (Figure 3a). Notably, HIV genome was not detected in tumor B-cells (data not shown), though they showed positivity for Tat (Figure 3b-c). To test whether DNMT up-regulation in B-cells was possibly resulting from a soluble Tat released from infected cells and taken up by uninfected B-cells, we used an *in vitro* model of B-cell lymphoma in which Tat was ectopically expressed either by exposure to soluble Tat or by transient and stable transfections. Relative expression of DNMT1, DNMT3a/b was then analyzed in our *in vitro* model by RT-qPCR and western blotting. Ectopic expression of Tat, either obtained by exposure to recombinant Tat or nucleofection, gave overlapping results, which showed that DNMTs are up-regulated following Tat ectopic expression, both at the mRNA and protein levels, as observed in primary tumors (Figure 4a-c).

### DNMT-regulating miRNAs are dysregulated in Tat-transfected cell lines

**Figure 5:**
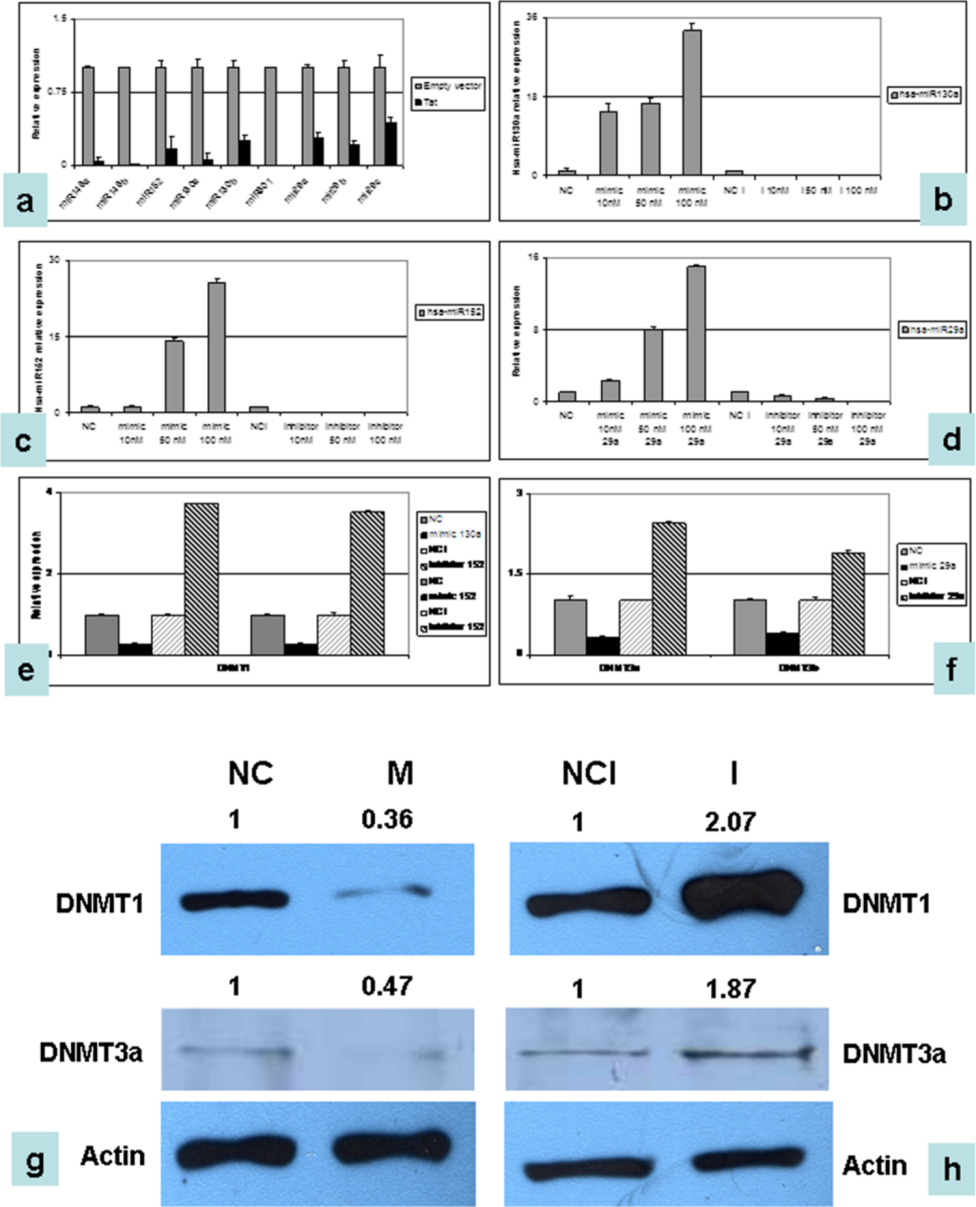
**Expression of DNMTs and DNMT-regulating miRNAs in Tat-positive vs. Tat-negative cells.** DNMT1 and DNMT3a/b-regulating microRNA expression was checked by RT-qPCR in Tat-positive vs. Tat-negative cells following transient transfections and exposure to recombinant protein **(a)**. Down-regulation of all miRNAs is observed in Tat-positive cells. The graph is representative of three different RT-qPCR experiments. Error bars represent standard deviation between duplicates. **(b-d)** Modulation of DNMT-regulating miRNAs was achieved by transient transfections of either mimics or antagomirs of the endogenous miRNAs at different concentration, and the effect on the expression of their respective miRNAs was monitored by RT-qPCR. DNMT1-regulating miRNAs (hsa-miR130a and hsa-miR152) are reported in **(b-c)**, whereas DNMT3a/b regulating miRNAs (hsa-miR29) are shown in **(d)**. **(e-f)** Relative expression of DNMT1 **(e)**, DNMT3a/b **(f)** was then evaluated following the ectopic modulation of these miRNAs. Up-regulation of the specific miRNAs results in the down-regulation of DNMTs, whereas increased expression of DNMTs is observed following miRNA inhibition. The graph is representative of three different RT-qPCR experiments (p < 0.05). Error bars represent standard deviation between duplicates. **(g-h)**: WB for DNMT1 and DNMT3a of cells transfected with hsa-miR152 (for DNMT1) and hsa-miR29 (for DNMT3a) mimics and inhibitors. NC: Mimic negative control; M: Either hsa-miR152 or hsa-miR29 mimic; NCI: Inhibitor negative control; I: Either hsa-miR152 or hsa-miR29 inhibitor. None of the tested antibodies for DNMT3b was suitable for WB analysis. Densitometric analysis results are reported.

We then checked whether Tat-dependent up-regulation of DNMTs relied on miRNA down-regulation, as in HIV-positive primary tumors. Our results demonstrated that the ectopic expression of the Tat protein is sufficient *per se* to determine DNMT-controlling miRNA down-regulation, both *in transient* (Figure 5a) and in stable Tat-transfected cells (data not shown). In addition, we used miRNA mimics and antagonists to modulate the expression of the endogenous miRNAs predicted to regulate DNMTs. Different concentrations for each mimic/antagonists were used, to assess the best dose–response effect (Figure 5b-d). The expression of DNMTs in cells transfected either with mimics or inhibitors was then checked, using mimics/inhibitors at the concentration proved to have the highest efficiency. Enhanced miRNA concentration resulted in decrease of DNMT1, DNMT3a/b whereas the inhibition of endogenous miRNAs resulted in their up-regulation, thus confirming regulation of DNMTs by the selected miRNAs in our cell model (Figure 5e-f). Western blotting confirmed modulation of DNMTs using mimics/inhibitors. In particular, ectopic over-expression of the selected miRNAs resulted in the down-regulation of DNMTs, whereas inhibition of the miRNA led to the consequent protein up-regulation (Figure 5g-h).

### Over-expression of DNMTs determines an increase of gene and microRNA methylation

**Figure 6:**
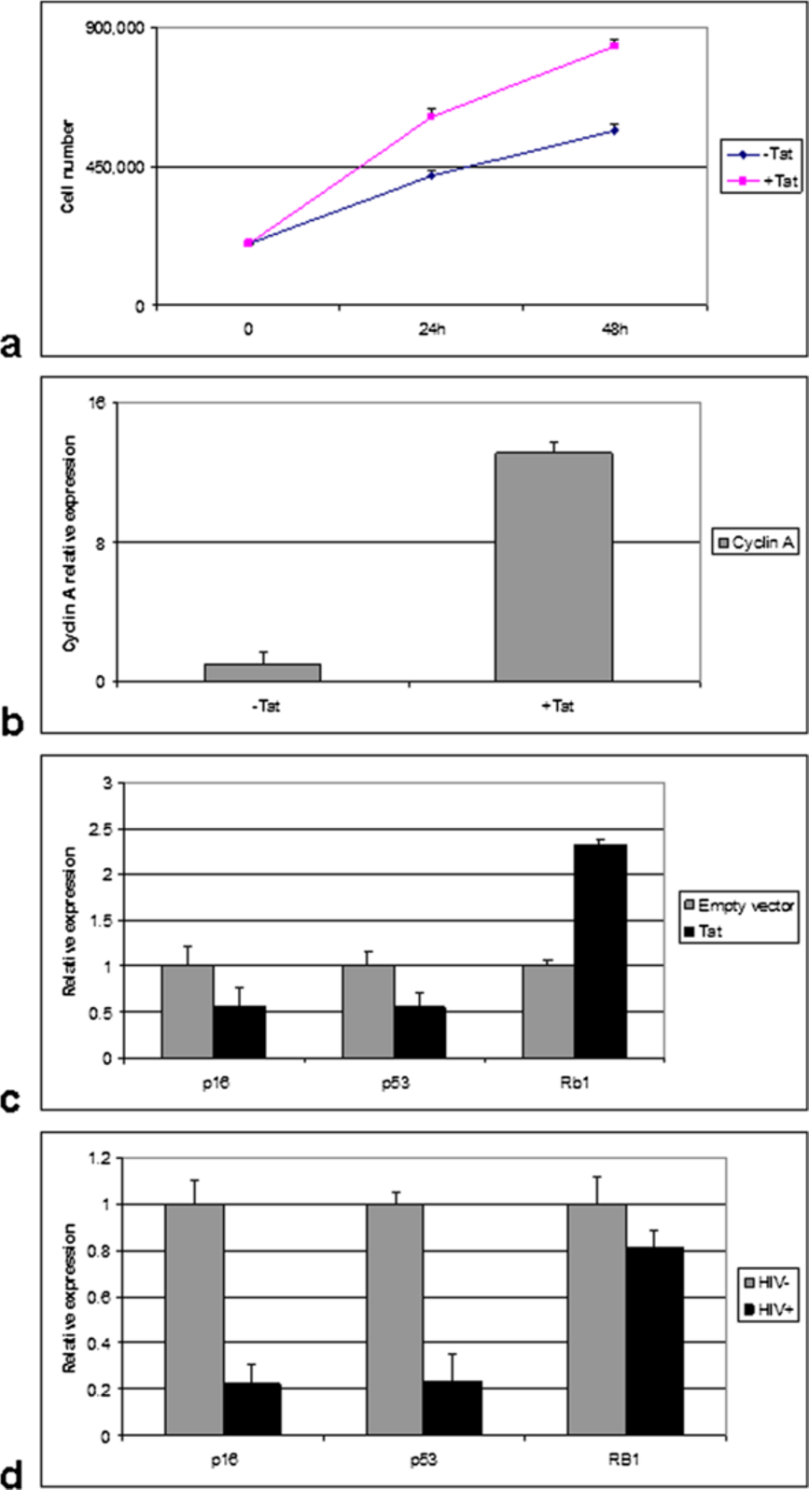
**Analysis of cell cycle regulators in Tat-positive vs. Tat-negative cells.** Cell proliferation in Tat-transfected vs. empty vector-transfected cells **(a)**. Tat-transfected cells show a higher proliferation rate, which is in line with the increased expression of cyclin A **(b)**, an S-phase specific marker. **(c-d)** Relative expression by RT-qPCR of *INK4/p16*, *TP53* and *RB1* in Tat-transfected cells **(c)** and in HIV-positive vs. HIV-negative primary tumors **(d)**. Down-regulation of *INK4/p16* and *TP53* is observed both *in vitro* and *in vivo*. The graph is representative of three different RT-qPCR experiments (p < 0.05). Error bars represent standard deviation between duplicates.

We have previously reported the physical interaction of Tat with the RB family of proteins, which results in the inactivation of their growth regulatory proteins [21, 38]. Therefore, we monitored cell growth in Tat-transfected cells and observed an enhanced cell proliferation (Figure 6a), which may depend on Tat-mediated alteration of the G1/S transition of the cell cycle, through the physical interaction of Tat with the pocket proteins [21, 38, 50]. Notably, the expression of cyclin A, which is a specific S-phase marker, was higher in Tat-expressing cells, which is consistent with the enhanced proliferation rate in Tat-positive cells (Figure 6b). Subsequently, we checked the expression of *RB1*, *INK4/p16* and *TP53*, crucial cell cycle regulators, in Tat-transfected cell lines. Our results demonstrate that the ectopic expression of Tat resulted in down-regulated expression of *INK4/p16* and *TP53*, whereas no difference was instead observed for *RB1* (Figure 6c). These *in vitro* results were then confirmed in HIV-positive primary tumors, by RT-qPCR (Figure 6d).

**Figure 7:**
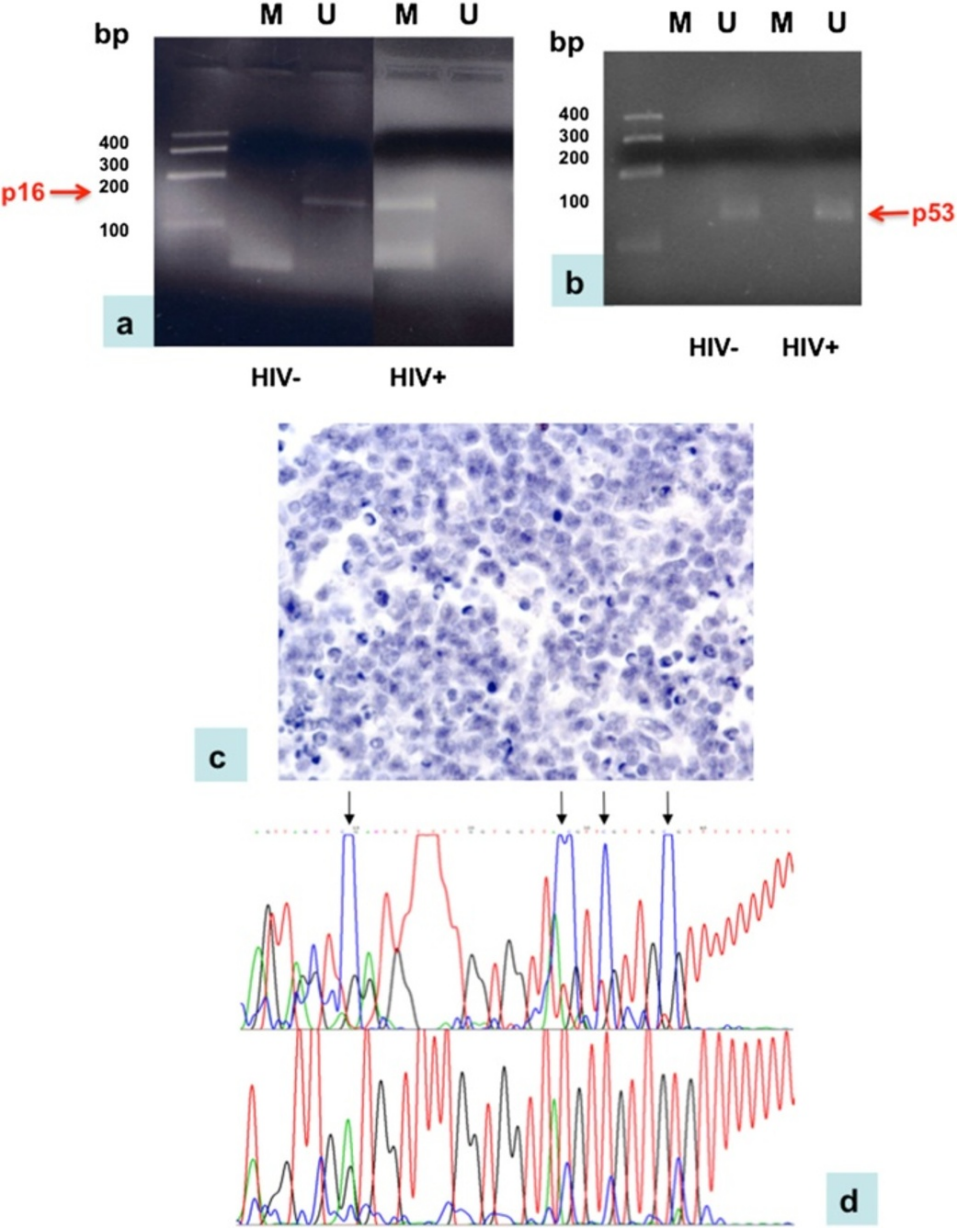
**Methylation analysis of DNMT-dependent miRNAs and cell cycle regulatory genes in Tat-positive vs. Tat-negative cells and primary tumors.** Methylation analysis of *INK4/p16* **(a)** and *TP53* **(b)** in HIV-positive and -negative primary tumors. M indicates the product using the methyled primers, U indicates the product using the unmethyled primers. Amplification of *INK4/p16* resulted in a band lower than 200 bp, whereas the specific product for *TP53* was about 100 bp. The specific product for *INK4/p16* was obtained only using unmethyled primers in HIV-negative samples, whereas only methyled primers resulted in a *INK4/p16* product in HIV-positive tumors, which is indicative of *INK4/p16* methylation in HIV-positive tumors. No differences were observed for *TP53* between HIV-negative and HIV-positive tumors, as the amplified product was obtained only using unmethyled primers, indicating that no methylation is detectable for *TP53* in neither of the two tumor types. Arrows indicate the presence of specific products for both genes. **(c)** IHC for p16 in HIV-positive tumors; **(d)** Sequence analysis of hsa-miR-148a in HIV-positive and HIV-negative primary tumors. Arrows indicate the methyled C that are not converted to U in HIV-positive tumors.

To verify whether down-regulation of *INK4/p16* and *TP53* was a consequence of promoter hypermethylation, DNA extracted from Tat-transfected cells and primary tumors was modified by bisulfite treatment, followed by MSP and BSP analyses. *INK4/p16* was found hypermethylated both in Tat-transfected cells as well as in HIV-positive tumors, whereas no methylation was detected for *TP53* (Figure 7a-b), whose down-regulation may possibly depend on genetic alterations. Decreased protein levels for p16 were also detected by IHC in HIV-positive primary tumors, in line with the *INK4/p16* gene hypermethylation (Figure 7c). Hypermethylation was also checked for those miRNAs containing CpG islands. In particular, BSP and MSP analyses identified partial methylation for hsa-miR148a and hsa-miR152 (Figure 7d), both in primary tumors and Tat-transfected cell lines (Table 5), whereas it was not possible to design appropriate primers for hsa-miR130a, due to the length of the product.

**Table 5:** **Analysis of methylated CpG sites for hsa-miR-148a and hsa-miR-152 in HIV-positive vs. negative primary tumors**

	HIV ^+^	HIV ^-^
**Hsa-miR-148a**		
Total CpG islands	4	4
Methylated CpG	4	0
Unmethylated CpG	0	4
**Hsa-miR-152**		
Total CpG islands	30	30
Methylated CpG	25	11
Unmethylated CpG	5	19

**Figure 8:**
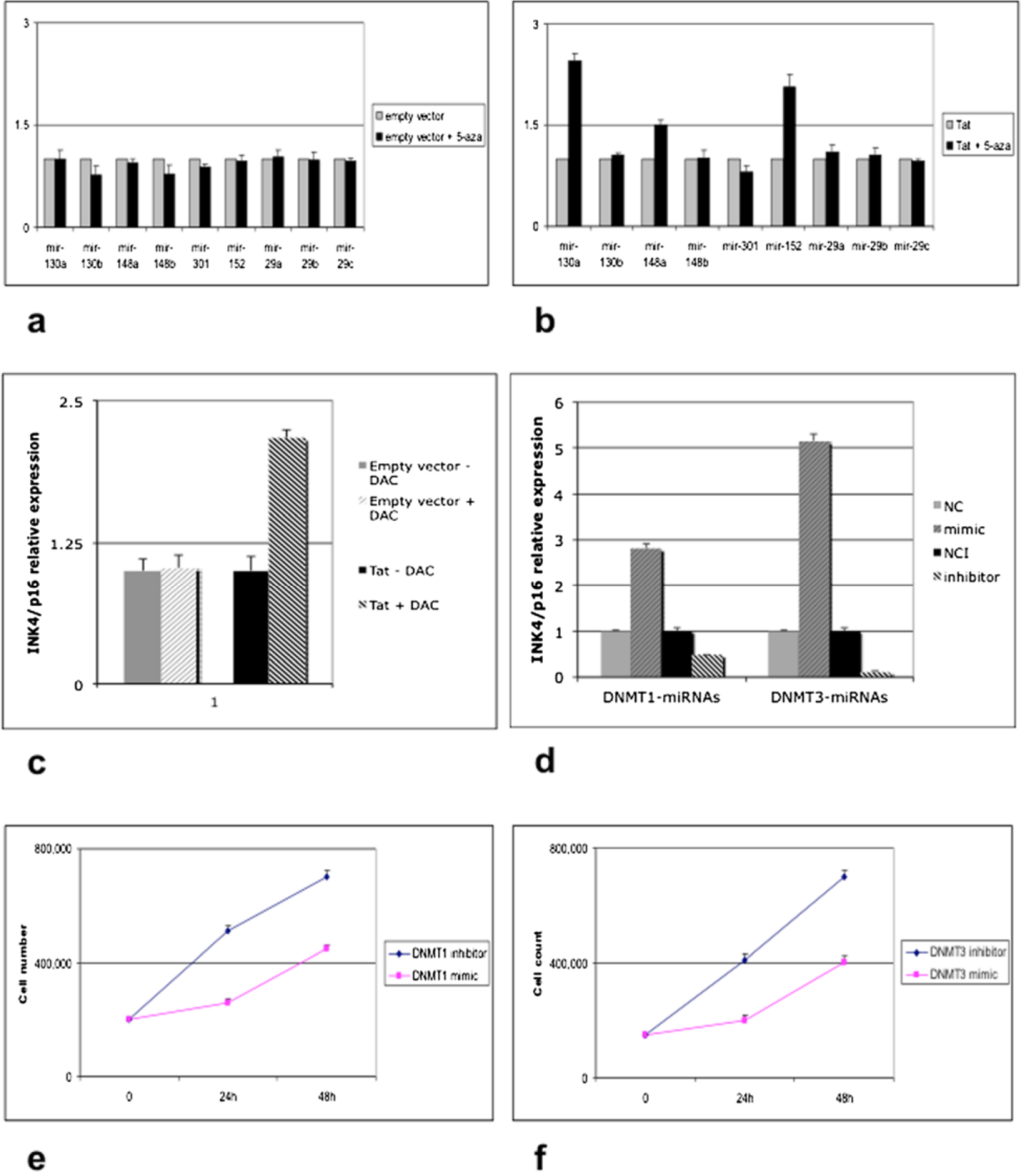
**Modulation of DNMT-regulating miRNAs affects the expression of** ***INK4/p16*** **and consequent cell growth control.** Treatment with 5-Aza in **(a)** empty vector-transfected and **(b)** Tat-transfected cells. Treatment with 5-Aza induces the expression of miRNAs containing CpG islands in Tat-transfected cells, indicating that down-regulation of these miRNAs depends on hypermethylation. **(c)** Treatment with 5-Aza was also able to restore the expression of *INK4/p16* in Tat-transfected cells, thus suggesting that silencing of *INK4/p16* is due to hypermethylation. **(d)** Ectopic modulation of DNMT-regulating miRNAs was achieved using synthetic mimic or inhibitors. Relative expression of *INK4/p16* was then checked by RT-qPCR. A decreased expression of the gene was observed following inhibition of miRNAs, which leads to up-regulation of DNMTs and consequent hypermethylation of target genes. Conversely, increased expression of endogenous miRNAs, which leads to downregulation of DNMTs, results in over-expression of *INK4/p16* (p < 0.05). NC: Mimic negative control; M: Either hsa-miR152 or hsa-miR29 mimic; NCI: Inhibitor negative control; I: Either hsa-miR152 or hsa-miR29 inhibitor. **(e-f)** Cell proliferation analysis in cells transfected with miRNA mimics/inhibitors for DNMT1 **(e)** and DNMT3a/b **(f)**. Inhibition of the endogenous miRNAs enhances cell growth.

5-Aza treatment was then performed in Tat-transfected cell lines, to further confirm that miRNA down-regulation was dependent on hypermethylation. Following 5-Aza treatment, miRNA expression level was checked by RT-qPCR. Our results demonstrated re-expression of miRNAs containing CpG islands (Figure 8a-b), whereas no change in the expression was instead observed for those miRNAs which do not contain CpG islands, as expected (Figure 8a-b). In addition, treatment with 5-Aza restored the expression of *INK4/p16* in Tat-transfected cells, thus confirming silencing of the gene through hypermethylation in the presence of Tat (Figure 8c). To further confirm that down-regulation of *INK4/p16* in the presence of Tat was due to miRNAs regulating DNMTs, we modulated the expression of the endogenous DNMT-regulating miRNAs using synthetic mimics and antagomirs, and consequently evaluated the expression level of *INK4/p16*. Our results demonstrated that the expression level of *INK4/p16* varied according to the modulation of DNMT-regulating miRNAs. In particular, inhibition of the endogenous miRNAs, which determines DNMT over-expression, resulted in a decreased expression of *INK4/p16*, which is consistent with DNMT-dependent hypermethylation. Conversely, ectopic up-regulation of the endogenous miRNAs using synthetic mimics led to the up-regulation of *INK4/p16*, as a consequence of a reduced DNMT expression (Figure 8d).

As DNMT-mediated silencing of *INK4/p16* through methylation may affect cell proliferation, due to the inactivation of its growth arrest properties, we monitored whether modulation of DNMT-regulating miRNAs through mimics/inhibitors may eventually affect cell growth. Our results show that inhibition of endogenous DNMT-regulating miRNAs through antagomirs results in an increased cell proliferation. Inhibition of endogenous miRNAs may affect cell proliferation through the up-regulation of DNMTs and consequent silencing of *INK4/p16*, which results in the abolishment of cell growth arrest. Conversely, DNMT down-regulation through miRNA mimics determines an opposite effect on cell growth (Figure 8e-f).

## Discussion

Tumor viruses has been implicated in the etiology of many cancers including malignant mesotheliomas, non-Hodgkin's lymphoma and tumors of the bone, brain and urinary tract [51–53]. The most commonly explored role for viruses in cancer involves the expression of viral oncogenes, such as polyomavirus large T-antigens (TAg), adenovirus E1a and E1b, and papillomavirus E6 and E7 [54]. These proteins share the ability to interact with and inactivate the pocket protein family (pRb, p107, p130) and/or the p53 tumor suppressor [55–58]. This results in the activation of the cellular DNA replication machinery needed to replicate the viral genome and promotes increased cellular proliferation, delayed differentiation, and often malignant transformation [59].

HIV infection is often associated with the onset of malignant lymphomas, 95% of which are of B-cell origin. Some of them, as Burkitt lymphoma, may arise in immunocompetent patients, even before the AIDS manifestation. Due to the high CD4^+^ cell number of these patients, it is reasonable to hypothesize that malignant transformation in these cases may not be a consequence of the immunodepression of infected individuals, suggesting that HIV itself may be involved in driving the transformation process. The virus encodes for many proteins and viral microRNAs, using which it may compete with cellular proteins/RNAs, thus disturbing the physiological regulation of the host cell. As most transformed B-cells do not contain the virus, some other mechanisms and/or viral factors may be involved. Among these, the most reliable candidate is the Tat protein, as it may function as a soluble effector, being released from infected cells and taken up by uninfected B-cells, in a biologically active form. Our previous studies demonstrated that HIV-positive B-cell lymphomas may be positively stained by an anti-Tat antibody [21] and again here we show positivity for Tat in our set of B-cell tumors. Therefore the endocytosed Tat may directly exert its biological functions in uninfected B-cells.

The ability of Tat to act directly on B-cells and differentially modulate the B-cell response of naïve/memory and germinal center (GC) B-cells has been previously reported [19]. Tat-mediated induction of GC B-cell proliferation might therefore contribute to promote HIV-associated follicular hyperplasia, autoimmune disorders and B-cell malignancies. The effects of Tat are likely to impact on the early stages of the cell cycle, before the G1 to S phase transition, and on B-cell differentiation, rather than affecting isotype switching [19]. In addition, Tat has been shown to bind to the pocket proteins, thus interfering with control of cell growth [21, 38, 50], which may eventually result in transformation.

However, while genetic perturbations have been shown to play key roles in viral transformation, epigenetic modifications such as DNA methylation may also play important roles during viral infection and transformation, as viral infection affects *de novo* methylation and transcription of cellular genes as well [60–62].

In this paper we have analyzed the expression of a particular class of proteins, the DNA Methyl Transferases, which epigenetically regulate gene expression, as HIV-1 has been reported to induce the expression of DNMT1 *in vitro*, which has a key role in the maintenance of DNA methylation, and epigenetically regulate the expression of several genes [29]. Over-expression of DNMT1 correlates with increased genomic methylation [30] and has been associated with miRNA dysregulation in liver cancer, in which DNMT1 over-expression is induced by IL-6 over-production [28]. Here, we show that HIV enhances the expression of DNMTs involved in both the basic and the *de novo* methylation. Such up-regulation relies on the down-regulation of specific miRNAs predicted to regulate DNMTs, in primary tumors of aggressive B-cell lymphomas, compared to HIV-negative tumors and normal tissues. The increased expression of DNMTs could result in an altered pattern of methylation of target genes/miRNAs in HIV-positive subjects. Noteworthy, reduction of the global methylation has been recently reported in HIV-positive subjects following HAART treatment [63], thus supporting the finding that HIV is able to increase global DNA methylation. As the HIV genome has not been detected in the tumor tissues of the aggressive B-cell lymphomas we analyzed, we hypothesized that Tat, released in a soluble form from infected cells, could contribute to malignant transformation of these cases. To test this hypothesis, we used an *in vitro* model obtained by ectopic expression of Tat in B-cells, either by cell transfections or exposure to recombinant Tat. In line with our *in vivo* results, we observed that the ectopic expression of Tat was able to induce overexpression of DNMTs based on down-regulation of DNMT-regulating miRNAs, pointing at a direct role for Tat in regulating DNMT expression.

Based on the growth capability acquired by Tat-transfected cells, which may depend on silencing of cell cycle progression inhibitory genes, we have then analyzed whether key cell cycle regulatory genes, as *INK4/p16*, *TP53* and *RB1*, may be possibly silenced through methylation, thus leading to the loss of the G1/S control [50], in Tat-transfected cells. Our results demonstrate a down-regulated expression of *INK4/p16* and *TP53*, in Tat-transfected cells, whereas no difference is observed for *RB1*. In addition, we show that the reduced expression of *INK4/p16,* following ectopic expression of Tat, was specifically due to hypermethylation, whereas no methylation was detected for *TP53*. 5-aza-2-deoxycitidine treatment restored the expression of down-regulated miRNAs, for which CpG islands have been described, thus suggesting that miRNA down-regulation Tat-transfected cell lines may depend on hypermethylation resulting from aberrant DNMT expression. The *in vitro* results were then confirmed in HIV-positive primary tumors. Interestingly, cell growth was affected by transfections of mimics/inhibitors, as inhibition of the endogenous miRNAs resulted in a higher proliferation rate. This may be due to the up-regulation of DNMTs and consequent *INK4/p16* silencing through methylation, which removes the control on cell growth and speeds up cell proliferation.

Transcriptional inactivation of DNMTs has been recently reported to occur by Rb proteins and this effect was shown to be reversible by Rb-inactivating viral oncoproteins such as the T-antigen [32–36]. In particular, decreased DNMT expression has been linked to the activity of the *RBL2* gene product [37]. Notably, we have previously demonstrated that Tat is able to inactivate RBL2/p130 through a physical binding [21, 38]. This may represent an intriguing mechanism through which Tat up-regulates DNMT expression in B-cells of infected patients by inactivating *RBL2* activity.

**Figure 9:**
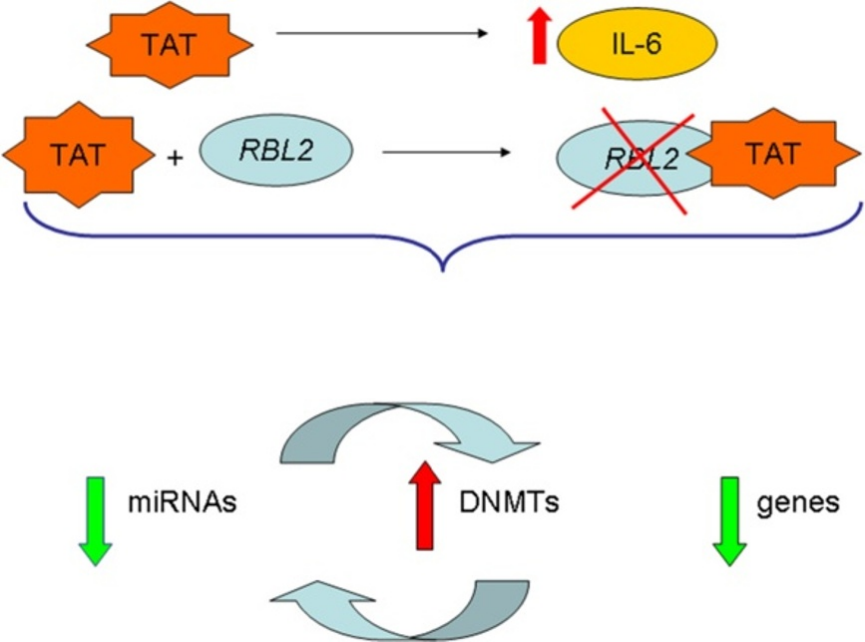
**A model for Tat-mediated lymphomagenesis.** DNMT aberrant activity may be induced by both IL-6 and RB-protein inactivation. As Tat is able to induce both IL-6 expression and *RBL2*-gene product inactivation, we hypothesize that Tat may induce DNMT up-regulation through these mechanisms, which results in the aberrant methylation of genes and miRNAs.

As Tat is also able to induce the expression of IL-6, which in turn determines aberrant DNMT1 activity, we may propose a model for Tat-mediated transformation in B-cells of HIV-infected patients, who have high levels of IL-6 (Figure 9). It is reasonable to hypothesize that a soluble form of Tat, released from infected cells within the B-cell tumor, is taken by uninfected B-cells. This biologically active Tat is now able to modulate the expression of several genes, including IL-6 [25]. The over-production of IL-6, together with the Tat-mediated inactivation of *RBL2*, could be then responsible for the up-regulated expression of DNMTs, which determines an aberrant methylation pattern of genes/microRNAs. Among these, hypermethylation of *INK4/p16* may be crucial, as its silencing would lead to the loss of control on a key cell cycle restriction point, as suggested by an enhanced cell proliferation, which may then confer cells a growth advantage and eventually result in malignant transformation.

## Conclusions

The Tat-dependent modulation of DNA Methyl Transferases provides an attractive mechanism through which it can restore and maintain methylation of critical genes in HIV-infected individuals.
